# Risk factors and immunological pathways for asthma and other allergic diseases in children: background and methodology of a longitudinal study in a large urban center in Northeastern Brazil (Salvador-SCAALA study)

**DOI:** 10.1186/1471-2466-6-15

**Published:** 2006-06-23

**Authors:** Mauricio L Barreto, Sergio S Cunha, Neuza Alcântara-Neves, Lain P Carvalho, Álvaro A Cruz, Renato T Stein, Bernd Genser, Philip J Cooper, Laura C Rodrigues

**Affiliations:** 1 Instituto de Saúde Coletiva, Universiade Federal de Bahia, Salvador, Brazil; 2 Instituto de Ciências da Saúde, Universidade Federal de Bahia, Salvador, Brazil; 3 Centro de Pesquisas Gonçalo Moniz – FIOCRUZ, Salvador, Brazil; 4 Centro de Enfermidades Respiratórias, Faculdade de Medicina, Universidade Federal de Bahia, Salvador, Brazil; 5 Department of Pediatrics, School of Medicine, Pontifica Universidade Católica, Porto Alegre, Brazil; 6 Instituto de Microbiologia, Universidad San Francisco de Quito, Quito, Ecuador; 7 Department of Epidemiology and Populations health, London School of Hygiene and Tropical Medicine, London, UK

## Abstract

**Background:**

The prevalence of asthma and allergic diseases has increased in industrialised countries, and it is known that rates vary according whether the area is urban or rural and to socio-economic status. Surveys conducted in some urban settings in Latin America found high prevalence rates, only exceeded by the rates observed in industrialised English-speaking countries. It is likely that the marked changes in the environment, life style and living conditions in Latin America are responsible for these observations. The understanding of the epidemiological and immunological changes that underlie the increase in asthma and allergic diseases in Latin America aimed by SCAALA studies in Brazil and Ecuador will be crucial for the identification of novel preventive interventions.

**Methods/Design:**

The Salvador-SCAALA project described here is a longitudinal study involving children aged 4–11 years living in the city of Salvador, Northeastern Brazil. Data on asthma and allergic diseases (rhinitis and eczema) and potential risk factors will be collected in successive surveys using standardised questionnaire. This will be completed with data on dust collection (to dust mite and endotoxin), skin test to most common allergens, stool examinations to helminth and parasites, blood samples (to infection, total and specific IgE, and immunological makers), formaldehyde, physical inspection to diagnoses of eczema, and anthropometric measures. Data on earlier exposures when these children were 0–3 years old are available from a different project.

**Discussion:**

It is expected that knowledge generated may help identify public health interventions that may enable countries in LA to enjoy the benefits of a "modern" lifestyle while avoiding – or minimising – increases in morbidity caused by asthma and allergies.

## Background

The prevalence of asthma and allergic diseases (rhinitis and eczema) has increased in industrialised countries in the last 2 decades [1-3]; in the same countries, there is tendency for the prevalence to be higher in urban than rural areas and to vary according to socio-economic status, although these findings are not consistent in all studies. Allergic disease results from a complex interaction between host genetics and environmental exposures [4] Since changes in host genetics occurring over so short a time scale is an unlikely explanation for the temporal trends in allergy prevalence, it is likely that the marked changes in environmental, life style and living conditions are known to have occurred in the last decades are responsible for these differences. Changes suggested t have contributed include urbanisation, better housing, diet and decreased exposure to infection early in life, and less exposures to helminths and other chronic infections.

Among environmental exposures that may have contributed to the trends in allergy prevalence, those that have attracted most interest are the fall in exposures to infectious diseases. The so-called "hygiene hypothesis" proposed that increases in the prevalence of allergic diseases were explained by improvements in living conditions, use of antibiotics and childhood vaccinations leading to a reduction in exposure to infections in early life [5]. Different immunological mechanisms have been postulated to mediate the biologic effects underlying the hygiene hypothesis. It has been hypothesised that exposure to viral and bacterial infections [5,6] and some vaccines [6-8] in early life, can enhance type-1 (Th1) immunity and the production of cytokines (i.e. IFN-g) that inhibit directly allergy-promoting type-2 (Th2) immune responses. The observation that individuals exposed to type-2 (Th2)-promoting helminth infections have a reduced prevalence of allergic disease has led to a reworking of this biological model. The new model hypothesises that early infectious diseases provide important regulatory signals for the establishment of robust immune regulation in early life, thereby preventing the development of the deregulated immunity associated with allergy.

Whatever the mechanisms involved the strong evidence that the temporal allergy trends are related to changes in lifestyle and living conditions raises the possibility that preventive interventions against allergic diseases can be identified. The understanding of the epidemiological and immunological changes that underlie the increase in asthma and allergic diseases will be crucial for the identification of novel preventive interventions.

International studies of the asthma prevalence have been conducted with the International Study of Asthma and Allergies in Childhood (ISAAC) study group using a standardised written questionnaire for self-reported asthma and respiratory symptoms. In these surveys, some Latin American settings (Brazil, Peru, Costa Rica, Paraguay and Uruguay) had very high prevalence rates, that were exceeded only by industrialised English-speaking countries [7]. These results appear inconsistent with the "hygiene hypothesis", given the poor socio-economic conditions and environmental problems present in most regions of Latin America (LA). LA countries suffer enormous societal disparities in wealth and development and with a large proportion of the population living in poverty [8,9]. It is not clear, therefore, why LA appears to have such high a prevalence of asthma, and there is scarce available information on how asthma and allergic diseases in LA vary according to socio-economic, urban-rural, environmental and life-styles factors. Studies in Brazil have shown that the prevalence of asthma is higher among non-white children and in those from low-income families [10,11].

The study described here is part of a larger ongoing research programme being conducted in Brazil (in the Northeastern city of Salvador) and Ecuador. Populations in both countries have experienced recent changes in living conditions and life style (urbanisation, improvement in sanitation). This research programme is funded by The Wellcome Trust through the programme of Major Awards to Centres of Excellence in Latin America and has adopted the name SCAALA (Social Changes, Asthma and Allergy in Latin America Programme). This paper specifically deals with the methodological aspects of the cohort study being conducted in Salvador, Brazil.

The Salvador cohort study has two main objectives. Firstly, to investigate the associations between the prevalence of asthma and other allergic diseases (rhinitis, atopic eczema) and potential risk factors that includes living conditions and early life and current exposures to infections, and secondly, to investigate how the association between environment factors, allergic diseases and markers of atopy (i.e. skin-prick test and total and specific serum IgE levels) are mediated by serum interleukins. In contrast, the study in Ecuador aims to study frequency of atopy and allergic diseases and exposure to potential risk factor in rural populations and in migrants from rural to urban areas; and examine how these may explain the risk of atopic diseases in migrants from rural to urban areas. This study will be described in a separate paper.

## Methods/Design

### Study site

Salvador, the capital of the State of Bahia, in the Northeast Brazil has approximately 2.5 million inhabitants, and is located in the poorest region in the country. Over 80% of the population is black or mixed-race (mulatto). There is a high degree of social inequality: GINI coefficient was 0.66 in 2000 [12]. The city currently presents high coverage of childhood vaccination (essentially 100% for neonatal BCG and measles-mumps-rubella vaccine, in 2003), water supply (95% of households with water supply in 2000) and sanitation (75% households with sanitation connection in 2000). The city of Salvador has several advantages as the site for this study. First, a ISAAC survey conducted in 1995 demonstrated a high prevalence of asthma: 27.1% and 12.5% of schoolchildren aged 13–14 years reported wheezing in the last 12 months [13] and asthma ever, respectively. Another study showed a prevalence of 10% among schoolchildren aged 12–16 years [14]. Second, significant improvements in living conditions have occurred in recent years. A sanitation programme was recently implemented (increasing the households connected to a safe sewage disposal from 30% to 70%) with subsequent reduction in the prevalence of intestinal parasitic infection and incidence of childhood diarrhoea [15]. Third, the health impact of such a sanitation programme has been assessed by a large epidemiologic study[15]; the participants of the evaluation of impact of the sanitation programme were recruited as the study population of the present study on allergic diseases.

### Study population and sampling

Children in this research project have been part of a study conducted earlier to evaluate the impact of the sanitation programme on the occurrence of childhood diarrhoea. This first study was originally designed to enrol three separate cohorts of children aged 0–3 years recruited from 24 small geographical areas selected to represent the population without sanitation in Salvador, called sentinel-areas [16,17] These three cohorts were recruited in 1997, 2001 and 2003, respectively. Within each selected area, a survey was conducted at baseline and the cohort was then followed up. Sampling and data collection procedures and instruments were the same in the 3 surveys and follow up making the study populations comparable. In each survey, children were randomly selected among those living in each area. Standardised questionnaires were applied during the baseline surveys to obtain data on demographic characteristics and living conditions of the families, sanitation, water supply and other characteristics of the households, pre-natal care, weight-at-birth, breastfeeding, attendance to kindergarten and vaccination status of children, nutritional status (anthropometry), characteristics of the neighbourhood and indoor environment. Children were then followed-up regularly for a period of one year from recruitment, forming thus the 3 cohort populations. During follow-up, children were visited every 2 weeks and data on occurrence of diarrhoea, cough, shortness of breath and fever as reported by the guardian was collected. Weight and height were measured using standard techniques and z-scores for height-for-age, weight-for-age and weight-for-height were calculated. A stool sample was collected and examined for parasitic infections. Details of the methodology and results of the first survey and cohort study have been published elsewhere [18-20].

To take advantage of these three cohorts (since detailed information for them had been collected on early life factors proposed as potential risk factors for asthma and other allergic diseases), the children who were aged 4–11 years and had complete follow-up information (e.g. stool examination conducted at 3 different time points) were selected for the present study.

### Study design

Follow-up during the current study on asthma and allergic diseases consists of two additional surveys to be conducted between 2005 and 2006, and again in 2008. The two surveys will assess children from the three previous cohort studies and will: (i) update information collected in the baseline surveys (ii) collect information on additional risk factors, (iii) collect stool samples for diagnosis of intestinal parasitic infections, (iv) collect blood samples for immunological analysis, (v) collect dust to measure allergen exposure, (vi) collect data on formaldehyde and particulate levels indoors and outdoors and (vii) collect data on asthma and allergic diseases (rhinitis, atopic eczema). The information collected during previous surveys and follow-up will be used in the present study to investigate early risk factors relating to infectious disease exposures and living conditions of the children from birth. All children from the previous 3 cohort studies were eligible to enter the present study.

### Sample size and study power

The total number of children recruited in the first resurvey with questionnaires completed was 1,445. Given this total number of children recruited, the magnitude of the prevalence rate ratio to be estimated with at least 80% of study power and 95% precision for selected characteristics were calculated by comparing proportions in STATA (command "sampsi"). This was done assuming an estimate of asthma prevalence of 10% [14], significance level of 5%, and the proportion of children exposed to specific risk factors, considering a loss rate of 10% and without making allowance for possible effect of clustering. Results for 4 selected characteristics are shown in Table 1.

### Main outcomes and case definition

Asthma, rhinitis and eczema will be defined according to the International Study of Allergy and Asthma in Childhood (ISAAC) [7]. Atopy will be defined as either a positive allergen skin prick test or the presence of allergen-specific IgE and allergy as the presence of clinical condition (asthma, rhinitis or eczema) in the presence of atopy [21]. Because no information on atopy or allergic diseases was collected previously, the frequency of allergic diseases will be measured as prevalence rates in the survey in 2005, and prevalence and incidence rates in the second survey in 2008 (incidence among those without disease in 2005); atopy will be estimated as prevalence in 2005 and rate of conversion/reversion in 2008.

### Data collection and instruments

The field work of the surveys in 2005 and 2008 involves 7 different activities, each conducted by different field teams and supervised by at least one of the co-investigators. Application of the main questionnaire, anthropometric survey, and stool and dust sample collection were completed in 2005. The activities were:

1. Questionnaire: mostly based on the ISAAC Phase II questionnaire [7], translated into Brazilian Portuguese. Some additional questions were included on environment, housing conditions, allergic diseases and potential risk factors, as described below. Questionnaire application was done by trained field workers, after piloting the questionnaire in children outside the study population but in the same target age. Medical assistance by the project in a referral outpatient care unit and by project doctors will be offered for all study children identified as having severe asthma.

2. An anthropometric survey with two independent measures of height and weight collected in a standardised way conducted by trained nutritionists, taking the mean value as the final measure, as recommended by WHO: z-scores for weight-for-age, weight-for-height and height-for-age were calculated using the EPINUT program (Epi Info 6.0; CDC, Atlanta, GA, USA) (details published elsewhere [20]).

3. Stool samples collected for helminths and parasites with two different samples for each child, 2 days apart. Stools were analysed using the gravitational sedimentation technique of Hoffman, Pons & Janner to detect helminth eggs, protozoan cysts and oocysts. Two slides were examined for each stool sample. Quantification of helminth eggs was performed using the Kato-Katz technique [22]. All children with positive results were treated with appropriate antiparasitic drugs (Albendazole and Tinidazole).

4. Dust samples collected using a residential vacuum cleaner (Eletrolux Professional, 1220 watts) containing a nylon 25 um micromesh sock filter [23]. The childrens' beds were aspirated for two minutes over a 1 m^2 ^area, adjacent to the head side. The filters were weighed before and after collection of the dust samples. Fibres and large particles were removed from the dust with forceps and the fine particulated dust samples were weighed, aliquotted as 100 mg samples and cryo-preserved at -20°C. Temperature and air humidity were recorded at the bedroom with a thermohygrometer (Minipa Ind & Co, São Paulo, Brazil).

5. Blood samples: An aliquot of 10 mL collected to measure (i) total and allergen specific IgE (anti-mite, and anti-cockroach), (ii) IgG antibodies to Hepatitis A virus, Herpes simplex, Herpes zoster, and Epstein-Barr viruses, *A. lumbricoides*, *T. trichura*, *Toxoplasma gondii *and *Toxocara canis*, and (iii) cytokines IFN-gamma, IL-13 and IL-10 and TGF-β in supernatant fluids collected from antigen-stimulated whole blood cultures.

6. Skin prick test (SPT) carried out by allergen skin prick testing of the right forearm of each children using extracts (ALK-Abello, São Paulo, Brazil) of *Dermatophagoides pteronyssinus*, *Blomia tropicalis*, *Blattella germanica*, *Periplaneta americana*, fungi, dog and cat epithelia. Saline and histamine was used as negative and positive controls, respectively. Wheal sizes will be read after 15 minutes and reactions will be considered positive if the mean of the two larger perpendicular diameters of the reaction is at least three millimeters superior to the mean of the two larger perpendicular diameters of the negative control reaction area.

7. Skin inspection for flexural dermatitis performed by trained observers using the ISAAC phase II protocol [24] will complement data on atopic eczema collected by questionnaire.

### Laboratory techniques

Aeroallergens will be quantified from dust samples by capture ELISA using commercially available kits (Indoor Biotechnologies, Virginia, USA) for the following aeroallergens: *Blomia tropicalis *Blo t 5, Dermatophagoides pteronyssinus Der p 1, Blatella germanica Bla g 2, Cat Fel d1, Dog Can f1Bacterial endotoxin and fungal b-glucan will be measured using the Limulus Amebocyte Lysate (LAL) assay (Cambrex Bio-Ciência do Brasil Ltda) following the manufacturers' instructions.

Heparinised whole blood will be cultivated at a dilution at 1:4 in RPMI (Gibco, Auckland, NZ) containing 10 mM glutamine (Sigma, St. Louis, MO, USA), 100 μg/ml of gentamicyn (Sigma, St. Louis, MO, USA), and stimulated with the following antigens: *Ascaris lumbricoides *(10 μg/ml); *Blomia tropicalis *(40 μg/ml); *Dermatophagoides pteronyssinus *(5 μg/ml). Pookweed mitogen (Gibco, Auckland, NZ) diluted at 1/1000 will be used as a positive control and media alone as negative control. House dust mites cultivated in fish food will be purified, lysed in pH 7.4 phosphate saline (PBS) with the use of a blender (Blender 51BL30; Waring Commercial, Torrington, Connecticut, U.S.A.). The *A. lumbricoides *antigen will be obtained by trituration of liquid nitrogen frozen adult worms obtained from a child treated with albendazole, in a blender as stated above in the presence of PBS. After centrifugation, the supernatants will be cryopreserved for storage before use. All antigens will be depleted of endotoxin by treatment with triton-114 (Sigma, St. Louis, MO, USA) and the protein contents will be determined by the Lowry method. Whole blood cultures will be cultivated at 5% CO2, for 24 hours for detection of IL-10 and for 5 days for the detection of IL-13, TGF-β and IFN-gamma. Cytokines in supernatant fluids will be measured using Pharmigen BD antibody pairs and recombinant standards (Pharmigen, San Diego, Ca, USA) by capture ELISA following the manufacturer's instructions. Cytokines will be estimated by interpolation of standard curves of recombinant standards.

Total IgE will be measured as follows: COSTAR high binding microassay plates (COSTAR, Cambridge, Me, U.S.A.) will be coated with 4 μg/ml of an anti- human -IgE antibody (PHARMIGEM, San Diego, CA, USA) overnight at 4C. Plates will be blocked with 0.15 M phosphate-buffered saline, pH 7.2 (PBS), containing 20% of dried skimmed milk (DSM) and 0.05% of Tween 20 (Sigma, St Louis, MO, USA) overnight, at 4°C. Sera to be tested and IgE antibody standards (RESEARCH DIAGNOSTICS INC, Flanders, NJ, USA). will be diluted 1:10 in PBS containing 10% of DSM and 0.05% of Tween 20 and incubated overnight at 4°C. A goat anti-human IgE-peroxidase conjugate (SIGMA, St Louis, MO, USA), and an anti-goat immunoglobulin-peroxidase conjugate (DAKO A/S, Glostrup, Denmark), will be diluted at 1:2000 and 1:10.000, respectively, and incubated for 60 minutes at room temperature. The results will be expressed in International Units (IU). A pool of parasite infected patients' sera will be used as positive control. Umbilical cord serum from a non-atopic and non-parasited mother will be used as negative control.

Determination of specific IgE serum concentration will be done for *Dermatophagoides pteronyssinus*, *Blomia tropicalis*, *Blatella germanica*, dog and cat epithelia main allergens using the RAST system (Pharmacia Diagnostics AB, Upsala Sweden).

Detection of anti-parasite antibodies. Anti-*A. lumbricoides *IgG4 will be detected by an indirect ELISA using wells of high binding microassay plates (COSTAR, Cambridge, Me, U.S.A.), sensitized with 20 μg/ml, of the parasite antigen as stated above, diluted in carbonate-bicarbonate pH 9,6 buffer. The sera will be diluted at 1:50 in PBS containing 10% skimmed milk and 0,1 % tween 20 (PBS/SM/T). The reaction will be detected using a biotinylated anti-human-IgG4/(SIGMA Chemical Co., San Louis, MO, USA), streptoavidin/peroxidase (PHARMIGEN., San Jose, CA, EUA) and H_2_O_2 _e OPD (MERCK & Co., Inc., White house Station, NJ, USA). The assay cut-off will be the mean plus 3 SD of negative controls (sera from people with 3 negative stool samples collected serially). Anti-*Toxocara *IgG antibodies will be detected using 20 μg/ml of larvae excretorial-secretorial antigen obtained by cultivation of the larva as described by De Savigny et al. [30] (The sera will be diluted at 1:100 in PBS/SM/T and pre-absorbed with *A. lumbricoides*; the development of the reaction will be done as described for *A. lumbricoides *IgG4 except for the conjugate that will be a biotinylated anti-human IgG. The assay cut of will be the mean plus 3 SD of negative controls (sera from upper middle class people, without history of dog and cat contact). Anti-*Toxoplama gondii *IgG and IgM will be detected using commercially available ELISA kits (Diamedix Corporation, Miami, USA), following the manufacturer's instructions. IgG antibody against the following virus will measured: Herpes simplex, Herpes zoster, Epstein-Bahr and Hepatitis A virus. Detection will be done using commercially available ELISA kits (Diamedix Corporation, Miami, USA), following the manufacturers' instructions.

### Main risk factors

Data for study variables were collected either (i) during the 3 previous baseline surveys and that will be updated in the 2 forthcoming surveys (2005 and 2008), or (ii) are new data to be collected in these 2 forthcoming surveys. Table 2 shows the data to be collected and the timing of data collection.

In the earlier cohorts no data was collected regarding asthma and hence incidence cannot be estimated in the first survey (2005). In the second survey (2008) a measure of incidence will be estimated reflecting new cases arising between 2005 and 2008. However, establishing asthma diagnosis in infancy is difficult. Additional information on occurrence of episodes of shortness of breath, fever and cough that are available for a year following the baseline surveys will also be used. This information may be more accurate than prevalence estimates estimated from data collected using a later questionnaire for asthma symptoms in infancy.

### Overview of plan of analysis

Statistical analysis will be conducted according to a conceptual framework defining a proposed causal pathway. Bivariate association analysis will be carried out by calculating prevalence ratios and 95% confidence intervals to measure the strength of the association between each potential risk factor and the outcomes of interest. At his stage, the main outcomes of interest are atopy (expressed as skin test positivity and level of IgE to allergen), asthma and allergy). Multivariate analysis will be taken out in several steps. First, within each level, multivariate data reduction techniques (e.g. principal component analysis) will be applied to summarise highly correlated variables to index variables (e.g. socio economic status, Th1- related immune response). Secondly, for each level, multivariate regression models will be fitted to estimate association parameters adjusted for confounding factors of the same level. In addition, multi-level modelling and robust variance estimation techniques will be used to adjust for intra-subject correlation due to repeated measures on the same individual and/or geographical areas. Finally, to address the complex inter-relationships between risk factors of different levels and outcomes according to our pre-defined conceptual framework, we will apply the following analysis strategies: i) A hierarchical effect decomposition strategy fitting a sequence of regression models each including different blocks of covariates, similar to an approach suggested by other authors [25]; ii) Log-linear models, an association analysis technique for multi dimensional contingency tables to estimate conditional association parameters (relative risks), conditional on the distribution of the risk factors on the higher levels [26,27]. For example, the probability of developing asthma will be estimated, conditional on a positive skin prick test, a high IgE level and environmental exposures. In addition, interaction parameters will be introduced to model how the risk factors on different levels influence each other (e.g. modelling the impact of environmental exposures on the effect of high IgE level); iii) Finally, sophisticated statistical techniques such as structural equation modelling and path analysis [28,29] will be used to model the multiple relationships defined in the conceptual framework simultaneously aimed to quantify direct and mediated effect components of the risk factors [30].

### Ethical considerations

Ethical approval was obtained from the Brazilian National Ethical Committee in 2004. Written informed consent was obtained from the legal guardian of each subject. It details all the procedures in the course of the project. All the clinical relevant results will be sent to the subjects parents, and specific recommendations will be done by a trained clinician after reviewing each case.

## Discussion

The SCAALA programme that includes the Salvador and the Ecuador project, is expected to provide important new information and contribute to our understanding of why the prevalence of asthma and allergies appear to be so high in urban settings in LA. The two studies have different objectives and designs, but they have similar conceptual frameworks and share expertise and operational resources. These projects require extensive co-ordination, involve expertise from several different disciplines (epidemiology, medicine, immunology, chemistry, nutrition, social sciences) and complex logistics (large-scale field work, laboratory work, standardisation of instruments and techniques, data base set up and linkage and advanced analysis). Study designs used include surveys and case-control studies making possible the comparison of prevalence and risk factors for asthma and other allergic diseases between urban and rural environments (the Ecuador study) and a longitudinal study of the effects of early life exposures of an urban population on asthma and other allergic diseases (the Salvador study). The laboratory component will explore the relative importance of different immunological mechanisms in mediating the effects of environment exposures (with emphasis on helminths and other infectious diseases of childhood) on the risk of asthma and allergic diseases. Statistical analysis will involve the use of strategies that link data from different levels (e.g. socio-economic, environmental, clinical and immunological factors) and will use advanced statistical techniques to deal with this complex framework

The Salvador study is the continuation of three different cohorts initiated several years ago with objective of studying the effects in changes in sanitation on the risk of infectious diseases The earlier data collected for these cohort studies, therefore, will provide important information that will allow the study to examine the importance of the 'hygiene hypothesis' in determining allergy risk in an poor urban centre of LA Theses cohorts provide prospective data over a period of 12 months from between birth and 3 years of age. Data was collected through home visits every 2 on diarrhoea, reported fever, cough and shortness of breath. Demographic, socio-economic, sanitation-related environmental data was collected also. Each child was weighed, height was measured, and a stool sample was collected for parasitologic assessment. Second, although data was collected at the individual level, the use of "sentinel areas" throughout Salvador made possible the collection of community level aggregate data and or census data

In summary, the proposed programme aims to clarify the social and biological mechanisms mediating the effect of population changes on the frequency of atopic disease in LA. Latin America is undergoing a rapid process of population change, including urbanization, migration, economic development and adoption of a "modern" lifestyle. Efforts to improve water supply, sanitation, rubbish collection and other hygienic measures are common to the different LA countries. It is expected that knowledge generated may thus help identify public health interventions that may enable countries In LA to enjoy the benefits of a "modern" lifestyle while avoiding – or minimising – increases in morbidity caused by asthma and allergies.

## Abbreviations

ISAAC: International Study of Asthma and Allergies in Childhood

SCAALA: Social Change, Allergy and Asthma in Latin America

## Competing interests

The author(s) declare that they have no competing interests.

## Authors' contributions

MLB conceived of the study, and participated in its design and coordination and lead the drafting of the manuscript, SSC participated in the design, coordinated the study, participate on the plan of analysis and helped draft this manuscript; NAN carried out the stool sample studies and the allergens studies; LPC carried out the immunological studies; AAC carried out all clinical investiagtion, RTS participated inthe drafting on the manuscript; BG lead the development of plan of analysis, PJC participated in the conception of the study design and plan of analysis, LCR conceived of the study, participated in its design and plan of analysis and helped to draft the manuscript. All authors read and approved the final manuscript.

## Pre-publication history

The pre-publication history for this paper can be accessed here:



## Figures and Tables

**Table 1:** Estimation of the sample size and power of the study

					Assuming study power of 80%		
					
Risk factors (all categorical values: 1 = exposed, 0 = not exposed)	Number of children with data on the risk factor		Proportion exposed (risk factor = 1)	Estimated number with asthma	Estimated prevalence in:		PRR^4^
					
	n	90%			not exposed	exposed	
(a)	(b)	(c)^1^	(d)	(e)^2^	(f)	(g)	(i)

**Measles vaccination **(unvaccinated over vaccinated)	1,407	1,266	0.75	126	0.08^3^	0.15	1.7
							
**Maternal smoking during pregnancy **(smoker over non-smoker)	1,412	1,271	0.14	127	0.09	0.17	1.9
							
**Sanitation connection**	1,409	1,268	0.52	127	0.07	0.13	1.7
							
**Maternal schooling **(illiterate and primary over higher schooling)	1,441	1,296	0.76	130	0.05	0.11	2.0

1-assuming a loss rate of 10% for the survey in 2005, it is (b) multiplied by 0.9;

2-assuming a asthma prevalence of 10%, it is (c) multiplied by 0.1;

3- unvaccinated;

4- prevalence rate ratio.

**Table 2:** Variables already collected and variables to be collected in the current survey

Study variables	Origin of the data	
	
	Previous baseline surveys and follow-up	Present survey 2005–2006^1^
**Demographic and socio-economic**		
Sex	X	
Birth date	X	X
Race/skin color	X	
On socio-economic condition:		
• parental schooling	X	X
• domestic goods	X	X
		
**Environmental factors**		
Sanitation and water supply	X	X
Presence of pets		X
Indoor smoking		X
Housing (e.g., floor, number of rooms)	X	X
Presence of allergens in dust (mites and cockroach)		X
Presence of endotoxin and mould in the house		X
Whether the house is located in an area of intense car traffic		X
		
**Psycho-social factors**		
domestic violence		X
Experience of discrimination		X
Resilience		X
		
**Maternal and family related factors**		
Maternal smoking during pregnancy	X	X
Asthma and other allergic diseases in the family		X
Number of pregnancies	X	X
		
**Child health**		
Prenatal Care	X	X
Weight at birth	X	
Breast-feeding duration	X	X
Vaccination (BCG, DTP, measles)	X	X
Whether attended kindergarten	X	X
Number of people who live in each household		X
Nutritional status	X	X
Global health status, as evaluated by the guardian	X	
Diarrhoea longitudinal prevalence	X	
Cough longitudinal prevalence	X	
Short of breath longitudinal prevalence	X	
Fever longitudinal prevalence	X	
Other infections (pneumonia, otites, causes for hospitalisations)	X	X
Use of antibiotics	X	X
		

**Specific Infections**		
Intestinal helminth and parasitic infections (stool examinations)	X	X
Serologic test to *A. lumbricoides*, *T. trichura*, *Toxopalsma. gondii*, *Toxocara canis*, hepatitis A and other virus		X
		
**Atopy**		
Skin prick test		X
Total and specific IgE titres to most important allergens		X
Antibody to mites and cockroach		X
		
**Immunological makers**		
IL-10, IL-13, TGF-β, IFN-Gamma		X

Note: the same data are planned to be collected in a survey scheduled to 2008.
